# K-anonymity decay in multi-turn clinical large language model conversations

**DOI:** 10.3389/fdgth.2026.1832168

**Published:** 2026-06-25

**Authors:** James Weatherhead, Azra Hasan, Jake Weatherhead, George Golovko, Bradley Grant, Juan David Garcia, Hugo Sebastian Certuche, Reuben Peter Powell, Jose Marri Abril, Peter McCaffrey

**Affiliations:** 1 Graduate School of Biomedical Sciences, The University of Texas Medical Branch at Galveston, Galveston, TX, United States; 2 Department of Pathology, The University of Texas Medical Branch at Galveston, Galveston, TX, United States; 3 Department of Computer Science, University of Pretoria, Pretoria, South Africa; 4 Department of Pharmacology & Toxicology, The University of Texas Medical Branch at Galveston, Galveston, TX, United States; 5 Transplant Division, Department of Surgery, The University of Texas Medical Branch at Galveston, Galveston, TX, United States

**Keywords:** clinical decision support, de-identification, HIPAA, k-anonymity, large language models, re-identification, shadow AI, synthetic data

## Abstract

Per-prompt de-identification, a commonly adopted practice for protecting patient privacy in clinical artificial intelligence (AI) conversations, may be insufficient to bound re-identification risk when the same patient is discussed across multiple conversational turns. Under the Health Insurance Portability and Accountability Act (HIPAA) Safe Harbor method, eighteen specified identifier categories must be removed from each disclosure independently, but this approach does not assess cumulative re-identification risk: successive turns reveal additional quasi-identifiers that progressively narrow the set of matching records, eroding the privacy protection that any single turn appeared to provide. We quantified this vulnerability by simulating progressive quasi-identifier disclosure, modeled on clinical case presentations, against a synthetic electronic health record cohort. Under progressive disclosure, 79.9% of simulated patients fell below the small-cell threshold (k<5) by the end of their disclosure sequence, with a median of seven disclosure steps to reach this threshold; when rare attributes were disclosed first, the median decreased to four steps. Even when no individual step disclosed a direct identifier, the cumulative quasi-identifier profile degraded k-anonymity below accepted safety thresholds. These findings suggest an operational limitation in per-prompt de-identification as applied to multi-turn clinical AI and large language model conversations: although HIPAA Safe Harbor includes a provision requiring no actual knowledge that remaining information could identify an individual, clinicians lack tools to assess cumulative re-identification risk in real time.

## 1 Introduction

Large language models (LLMs) have shown strong performance on medical knowledge benchmarks (1–4), coinciding with growing informal adoption by clinicians seeking decision support, documentation assistance, and diagnostic guidance. Recent surveys document widespread unsanctioned use of consumer artificial intelligence (AI) tools in hospitals and health systems, often called “shadow AI” (5). Physician use of AI in practice has roughly doubled every year since ChatGPT’s release: 38% in 2023, 48% in 2024, and 72% in 2026 per the American Medical Association (6), though this encompasses administrative uses alongside clinical decision support; a single-institution hospitalist survey similarly reported 66.7% adoption (7). A 21-country survey found that 76.2% of healthcare professionals had used ChatGPT (8), and in Elsevier’s 2025 survey of 2,206 clinicians across 109 countries, only 29% rated their institution as performing well on AI governance (9). Privacy is among the major ethical concerns identified in clinical LLM use (10). In practice, that use takes the form of multi-turn conversation: clinicians progressively disclose patient details (demographics, diagnoses, medications, procedures) to receive tailored clinical guidance. Many assume they adequately protect patient privacy by carefully omitting names, medical record numbers, and other direct identifiers from each prompt. Yet generative AI may amplify existing privacy vulnerabilities (11) in ways that Health Insurance Portability and Accountability Act (HIPAA) compliance alone does not address: cumulative quasi-identifier disclosure across conversational turns creates re-identification pathways that existing regulatory frameworks were not designed to evaluate, and LLMs themselves can now serve as deanonymization tools, autonomously linking pseudonymous profiles to real identities at scale (12).

Beyond user disclosure, privacy risks also arise from the models themselves. Generative LLMs can memorize and regurgitate portions of their training data (13), and medical LLMs fine-tuned on clinical text are especially exposed because training corpora often retain direct and quasi-direct identifiers even after de-identification (14). LLMs also retain information across sessions in ways that amplify cumulative exposure (15, 16), and agent-based systems can synthesize disclosed information with external records to reconstruct identities at scale (12). These training-time and inference-time mechanisms sit alongside the user-disclosure phenomenon this study examines, but are mechanistically separate.

The user-disclosure phenomenon is governed, in current practice, by HIPAA Safe Harbor de-identification (45 CFR §164.514), which permits disclosure of information stripped of 18 specified identifier categories (17). Under this framework, generalized demographics are considered sufficiently de-identified. For example, Safe Harbor requires that all ages over 89 be aggregated into a single category, and that geographic subdivisions include only the initial three digits of a ZIP code when the geographic unit contains more than 20,000 people. However, Safe Harbor does not guarantee protection against re-identification through quasi-identifier combinations. Safe Harbor also requires that the covered entity have no actual knowledge that the remaining information could be used, alone or in combination, to identify an individual [45 CFR §164.514(b)(2)(ii)]; a clinician who knows their patient’s identity and progressively discloses a narrowing quasi-identifier profile may fail to satisfy this requirement, though this has not been tested in the context of conversational AI. This represents a gap between per-category compliance and cumulative privacy protection. Safe Harbor does not address the Minimum Necessary Standard [45 CFR §164.502(b)], which requires limiting protected health information (PHI) disclosure to what is necessary for the stated purpose. The alternative Expert Determination method [45 CFR §164.514(b)(1)] explicitly requires statistical assessment of combinatorial re-identification risk, but to our knowledge, expert determination has never been applied to real-time conversational AI workflows.

K-anonymity, introduced by Sweeney, provides one formal approach to assessing this combinatorial risk (18). A dataset satisfies k-anonymity if every combination of quasi-identifier values appears in at least k records. Quasi-identifiers are attributes that, while not unique identifiers themselves, can be combined to identify individuals. Sweeney’s landmark working paper reported that 87% of the U.S. population could be uniquely identified using only ZIP code, gender, and birth date based on 1990 Census data (19), though subsequent peer-reviewed analysis using the same census estimated 61% (20); regardless of the exact percentage, both studies confirm that basic demographics enable substantial population identification. Healthcare analytics systems enforce suppression when k falls below risk thresholds: the Centers for Medicare & Medicaid Services (CMS) prohibits direct reporting of any cell containing a value of 1 to 10 (21); TriNetX thresholds aggregate query results so that no query directly returns a value smaller than 10 (22); the Centers for Disease Control and Prevention (CDC) suppresses rates and counts below 16 in U.S. Cancer Statistics (23).

While extensive research has examined re-identification risk in static datasets (24, 25), the sequential disclosure pattern characteristic of conversational AI remains largely unexplored: each conversational turn adds constraints that monotonically narrow the anonymity set, and once disclosed, information cannot be retracted. We measure k as one indicator of singling-out risk, not a complete privacy guarantee; l-diversity, t-closeness, and differential privacy are addressed in Section 4.4 (26, 27).

**Figure 1:**
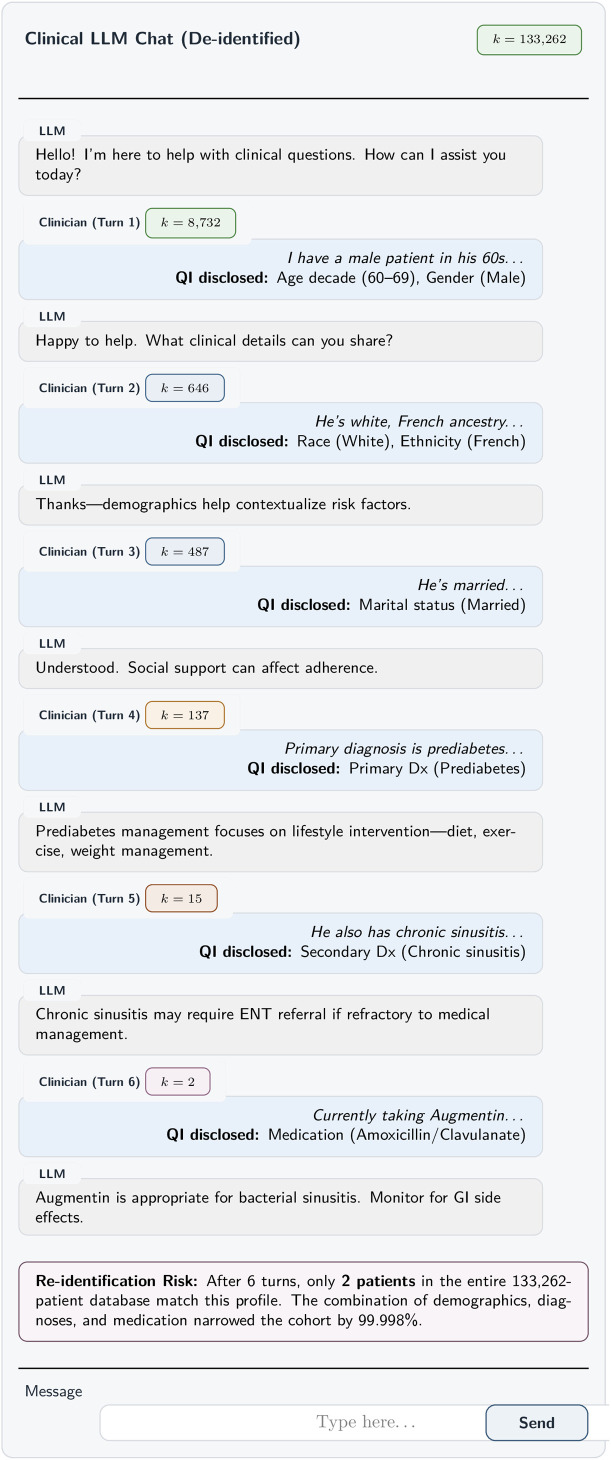
Illustrative clinical conversation demonstrating k-anonymity collapse. This figure depicts 6 compound conversational turns (where multiple quasi-identifiers are disclosed together, e.g., “male patient in his 60s”), which encompass 8 of the 11 individual disclosure steps tracked in our simulation. The k value decreases with each disclosure until reaching small-cell territory. All k-values computed from Synthea synthetic dataset (verified exemplar patient details in Supplementary Tables S5 and S6).

This study quantifies how k-anonymity degrades across multi-turn clinical LLM conversations. Throughout this paper, we distinguish between a conversational *turn* (a compound exchange in which a clinician may disclose several attributes at once, for example “male patient in his 60s”) and a *disclosure step* (the addition of a single quasi-identifier to the accumulated profile); a single turn may comprise multiple steps. We use “decay” to describe the gradual reduction of k-anonymity across disclosure steps, and “collapse” to describe reaching small-cell territory (k below threshold). The fundamental concern is this: a clinician may strip each conversational turn of the 18 Safe Harbor identifier categories (no names, no Social Security numbers, no addresses), yet the combination of these individually compliant disclosures across turns produces re-identification risk that no single turn would create alone. Figure 1 illustrates a simulated clinical conversation demonstrating this phenomenon. This study focuses on identity disclosure risk (determining which individual a record belongs to) rather than attribute disclosure (learning sensitive information about an already-identified individual), though both are relevant to clinical AI privacy. We hypothesized that such Safe Harbor-compliant quasi-identifiers, when accumulated across multi-turn clinical conversations, would reduce a majority of patients to small-cell territory (k<5). Understanding this phenomenon is necessary for developing privacy-preserving clinical AI systems.

## 2 Materials and methods

### 2.1 Threat model and scope

We assume an adversary with access to LLM conversation transcripts seeking to identify patients in a reference population. This “prosecutor model” requires: (1) conversation transcript access, (2) reference database, and (3) distinguishing information about a target. For services without a Business Associate Agreement (BAA), which contractually obligates the provider to safeguard PHI under HIPAA, the LLM provider itself represents a potential adversary with complete access to all conversation content.

### 2.2 Data source and limitations

We used SyntheticMass Version 2 (May 24, 2017) generated by the Synthea synthetic patient generator (28). The dataset comprises 133,262 synthetic patients with demographics, diagnoses (Systematized Nomenclature of Medicine: Clinical Terms [SNOMED CT]), medications (RxNorm), procedures, allergies, and encounters. This structured, coded representation reflects the subset of clinical information captured in electronic health record (EHR) problem lists, medication lists, and procedure logs; it does not include unstructured clinical narrative such as progress notes, nursing documentation, or allied health assessments, part of the broader unstructured corpus that comprises roughly 80% of medical data (29). Within that structured subset, Synthea has been validated: Chen and colleagues found it “quite reliable in modeling demographics and probabilities of services being offered in an average healthcare setting” (30), with noted limitations for body mass index (BMI) and heterogeneous post-service outcomes; the quasi-identifiers used in this study fall within the attribute classes Chen reports as reliably modeled.

#### 2.2.1 Ethical justification for synthetic data

We deliberately use synthetic data because publishing k-anonymity statistics on real hospital data would itself create attack vectors through membership inference and differencing attacks against aggregated health data (31). Synthetic data preserves realistic demographic distributions while mitigating re-identification risk for real patients (32).

#### 2.2.2 Data quality

After removing 197 malformed rows, 133,262 patients were included. Ages were computed relative to a reference date of December 31, 2017.

### 2.3 Quasi-identifier extraction

We extracted 23 quasi-identifiers from the Synthea dataset across six categories (demographics, conditions, medications, procedures, allergies, temporal attributes), of which 11 were selected for simulation; the remainder were excluded as redundant or derivable. Age was binned by decade; primary condition and medication were defined as the most frequently documented codes per record. We modeled single-value disclosure (one primary diagnosis, one primary medication, binary procedure and allergy indicators) rather than full lists (Supplementary Table S3). These choices bias toward underestimating k-decay (see Limitations).

### 2.4 Disclosure models

We implemented three disclosure ordering models to capture different conversation patterns. The Progressive Refinement model defines 9 compound turns, but because some turns disclose two quasi-identifiers simultaneously, the model unfolds across 11 individual disclosure steps (turn vs. step distinction defined in Section 1).

#### 2.4.1 Progressive refinement model

This model simulates workflows where clinicians begin with demographics and progressively add clinical details, grounded in established clinical communication conventions (33, 34). As reported by Brett and Goodman (35), a 2009 survey found that 62% of US medical schools acknowledged residents mentioned race in the first sentence of case presentations, and LLM interaction research confirms progressive disclosure patterns (36).

The model comprises 11 disclosure steps proceeding from demographics (age decade, gender, race, ethnicity, marital status) through clinical attributes (primary and secondary condition, primary medication, procedure and allergy indicators) to first encounter year (Supplementary Table S1); k is computed after each. Of sampled patients, 72.6% had complete data for all 11 steps; the remainder had 8 to 10 steps available (Supplementary Table S7). For patients with fewer attributes, the final k was carried forward, since k is monotonically non-increasing.

#### 2.4.2 Random ordering model

Discloses quasi-identifiers in random order to estimate variance in outcomes. For each of the 5,000 sampled patients, we conducted a Monte Carlo simulation, generating 30 random permutations, yielding 150,000 total simulation runs (Supplementary Table S8).

#### 2.4.3 Rarity-ordered model

Discloses quasi-identifiers in ascending order of population frequency (rarest first). This represents a worst-case scenario where distinctive attributes are revealed early (for example, disclosing a rare diagnosis before common demographics). While not typical clinical practice, this model establishes lower bounds on privacy protection (Supplementary Table S2).

### 2.5 K-anonymity computation

For each patient and disclosure step, we computed k as the count of records in the full 133,262-patient database satisfying all accumulated constraints. The value of k was computed against the full 133,262-patient population rather than the 5,000-patient sample to avoid artificially deflating equivalence class sizes. We tracked threshold crossings at k<5, the small-cell convention common in privacy research, and k<11, the CMS cell size suppression threshold (21).

### 2.6 Statistical analysis

We randomly sampled 5,000 patients for simulation using a fixed random seed for reproducibility (Supplementary Table S4). Sampling 5,000 of the 133,262 available patients reduced the Random Ordering model’s computational burden from approximately 4 million to 150,000 individual simulation runs while providing adequate statistical precision: with n=5,000, the 95% Wald confidence interval on the proportion reaching any given threshold has a half-width of approximately 1.4 percentage points, which is sufficient to estimate population-level privacy behavior at the precision meaningful for operational thresholds. K-anonymity was computed against the full 133,262-patient reference population, not the 5,000-patient sample (Section 2.5). For each model we computed proportion reaching threshold, median k at each step, and distribution of steps-to-threshold.

## 3 Results

### 3.1 Overall threshold crossing rates

Across all disclosure models, a substantial majority of simulated patients reached small-cell territory. Of 5,000 randomly sampled patients, 79.9% (3,994; 95% confidence interval [CI]: 78.8% to 81.0%) reached k<5 at some point during their disclosure sequence, while 86.2% (4,311; 95% CI: 85.2 to 87.2%) reached k<11. Of the 5,000 sampled patients, 64.6% (3,228) were uniquely identifiable (k=1) within the synthetic reference population by the end of their disclosure sequence. The identical proportions across models reflect that final k depends only on which quasi-identifiers are disclosed, not their order; ordering affects only how quickly thresholds are crossed.

Reaching k<5 does not equate to successful re-identification; the adversary still requires transcript access, a reference database, and distinguishing information. The simulation’s contribution is quantifying the rate of decay under clinically grounded disclosure patterns and establishing that operational suppression thresholds are crossed within conversation lengths characteristic of clinical consultations.

### 3.2 K decay patterns

**Figure 2:**
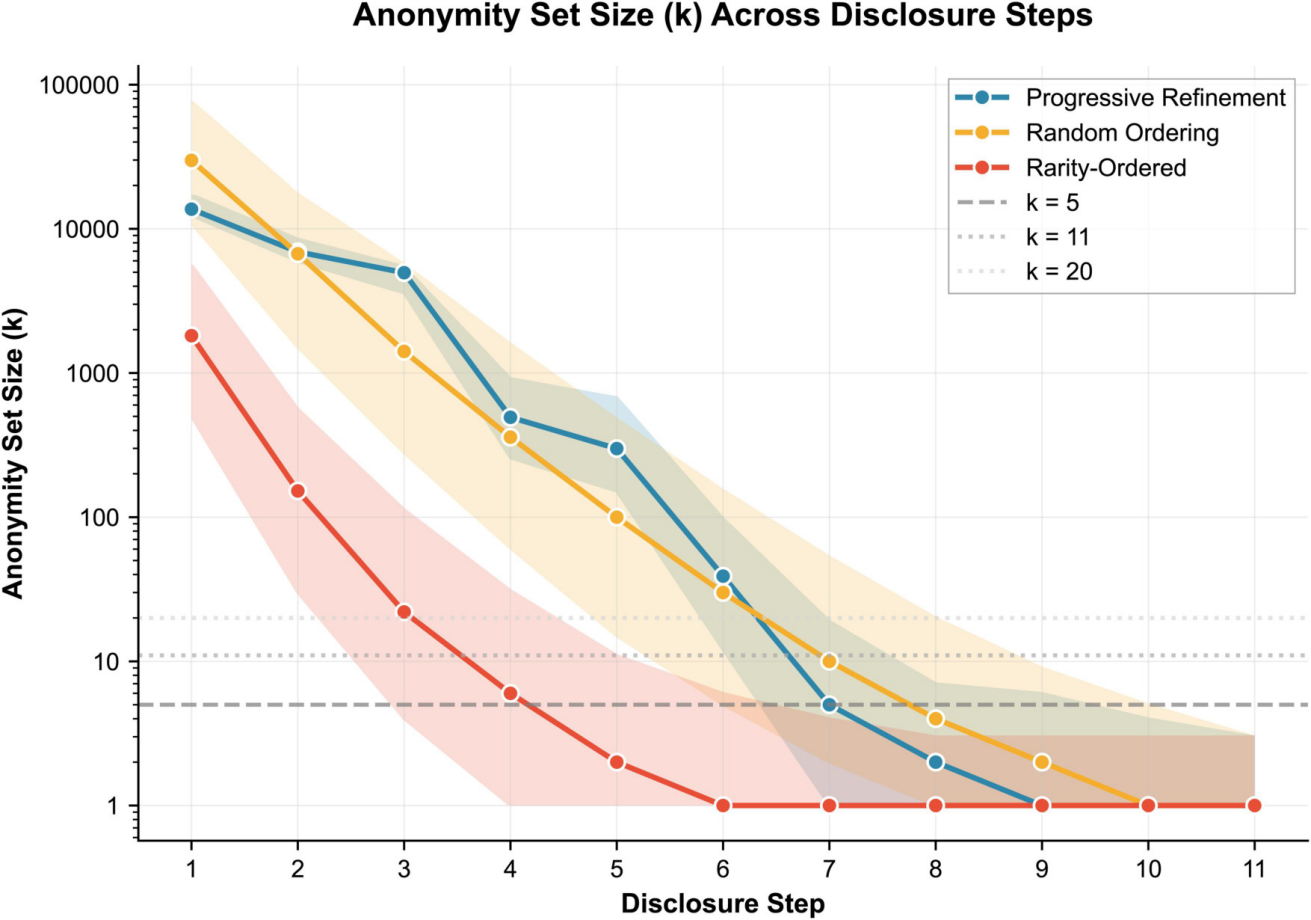
K-anonymity decay across disclosure steps. Median k (log scale) for Progressive Refinement (blue), Random (yellow), and Rarity-Ordered (red) models. Shaded regions: IQR. Dashed lines: k=5 and k=11 thresholds.

Figure 2 shows median k across disclosure steps. In the Progressive model, median k decreased from 133,262 to approximately 13,700 after Step 1 (age decade), with further reductions at ethnicity (Step 4) and primary condition (Step 6) producing the largest single-step decreases; by Steps 7 to 8, k reached single digits. In the Rarity-Ordered model, median crossing of k<5 occurred at step 4 (Supplementary Tables S1, S2, S8).

### 3.3 Time to threshold

**Figure 3:**
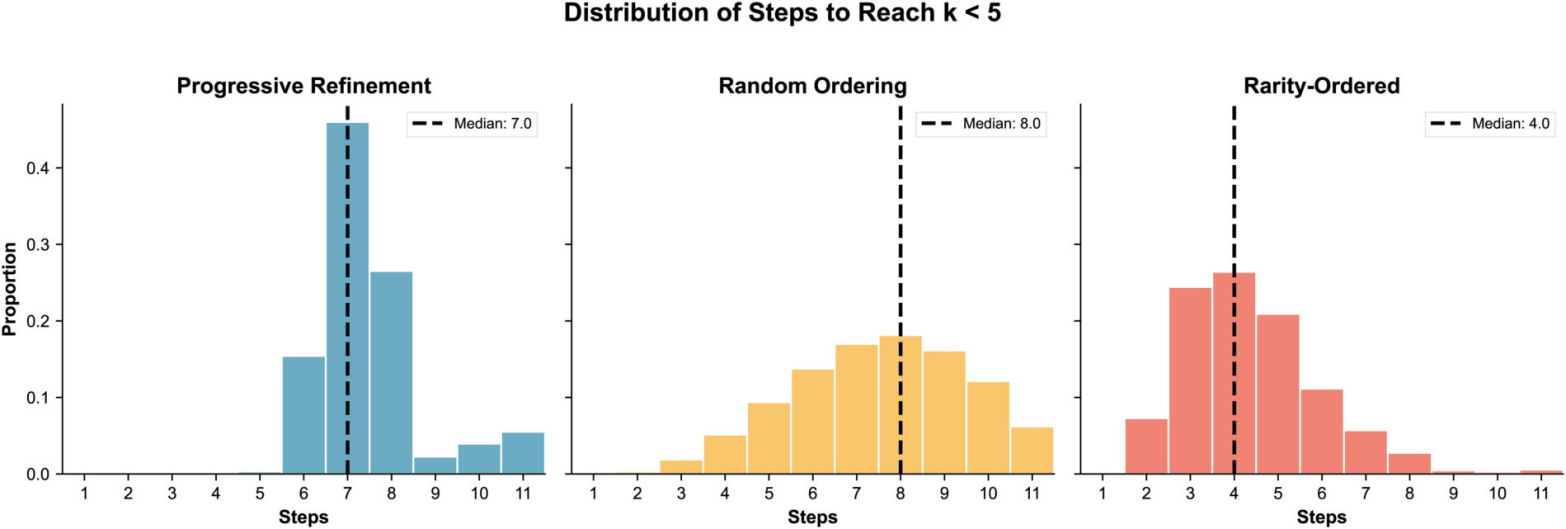
Distribution of disclosure steps to reach k<5. Progressive Refinement: median 7 steps (IQR: 7 to 8); Random Ordering: median 8 steps (IQR: 6 to 9); Rarity-Ordered: median 4 steps (IQR: 3 to 5).

Figure 3 shows steps to reach k<5. Progressive model: median 7 steps (interquartile range [IQR]: 7 to 8); Random: median 8 steps (IQR: 6 to 9); Rarity: median 4 steps (IQR: 3 to 5).

### 3.4 Threshold crossing dynamics

**Figure 4:**
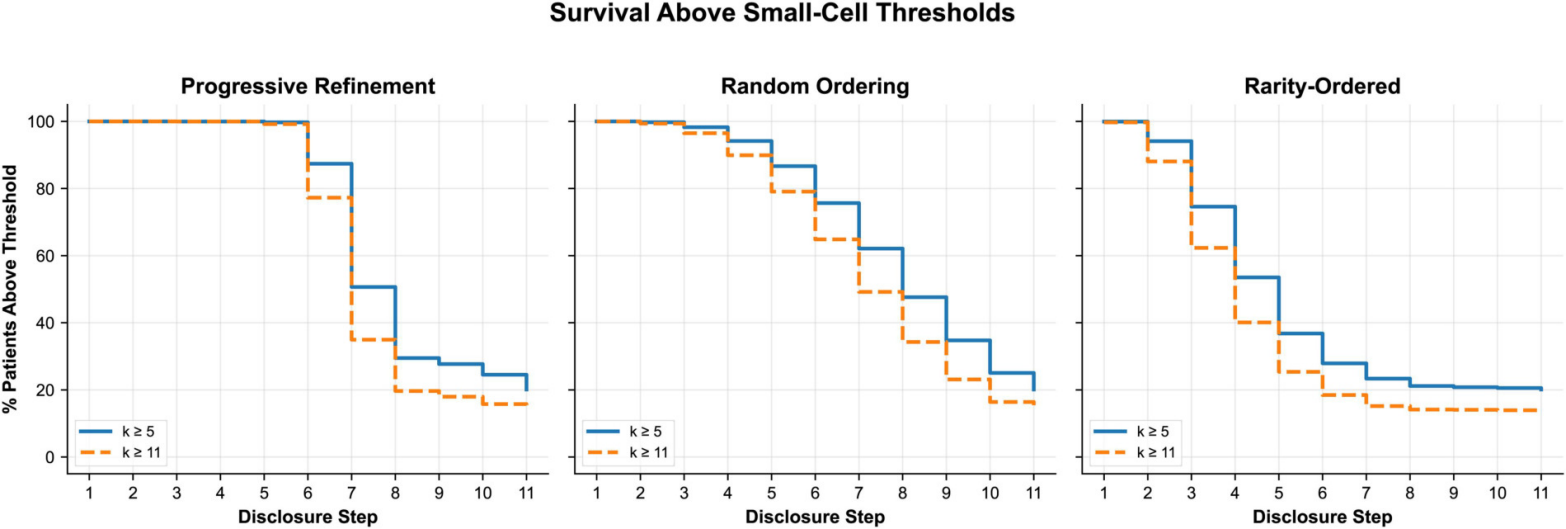
Proportion of patients remaining above k-anonymity thresholds across disclosure steps. Progressive Refinement (solid): 50% remain above threshold at step 7; Rarity-Ordered (dashed): approximately 50% remain above threshold between steps 4 and 5. Note: This visualization shows Proportion Safe (k≥ Threshold) and threshold crossing dynamics, not formal survival analysis; all patients complete their disclosure sequence without censoring.

Figure 4 shows the cumulative proportion remaining above threshold; the curves indicate small-cell territory is reached through steady accumulation rather than a single large disclosure.

## 4 Discussion

### 4.1 Principal findings

This simulation suggests that k-anonymity may degrade below accepted safety thresholds across multi-turn clinical LLM conversations: 79.9% of simulated patients reached small-cell territory (k<5) within their disclosure sequence, even though each turn contained only generalized attributes with none of the 18 Safe Harbor identifier categories. The re-identification risk emerges not from any non-compliant disclosure but from the steady accumulation of compliant ones. Clinicians using consumer AI tools without BAA coverage may believe they protect patients by omitting names and medical record numbers (MRNs), yet the cumulative quasi-identifier profile they build across turns can reduce anonymity below accepted thresholds; per-turn Safe Harbor compliance does not, by itself, confer cumulative privacy protection.

Safe Harbor’s “actual knowledge” clause [45 CFR §164.514(b)(2)(ii)] addresses cumulative risk in principle but requires clinicians to assess combinatorial re-identification risk intuitively and in real time, with no operational tools to do so. A clinician who has disclosed seven quasi-identifiers may have crossed into small-cell territory without awareness; the regulatory standard exists but is unenforceable absent monitoring tools.

Whether that gap amounts to a formal HIPAA violation turns on how Safe Harbor is read. Under a strict reading [45 CFR §164.514(b)(2)], each turn stripped of the 18 identifier categories is compliant. Under a combined reading that incorporates the actual knowledge clause, a covered entity aware of how cumulative disclosures narrow the anonymity set may fail the requirement of having “no actual knowledge that the information could be used alone or in combination…to identify an individual.” To our knowledge, no enforcement action or Department of Health and Human Services guidance has addressed this question for conversational AI. The Expert Determination pathway [45 CFR §164.514(b)(1)], by contrast, requires an expert with “appropriate knowledge of and experience with generally accepted statistical and scientific principles and methods” to apply those methods in a formal, repeatable, documented process and certify that re-identification risk is very small (37); this framework was developed in the context of clinical-trial dataset sharing, with threat models that assume a bounded, anticipated recipient of a released dataset, and no published methodology operationalizes Expert Determination on a per-prompt basis for conversational AI, where legal scholars have argued that “chatbots cannot comply with HIPAA in any meaningful way despite industry assurances” (38). Disclosure ordering affects collapse rate but not final outcome: progressive clinical workflows reach k<5 at a median of 7 steps, while rarity-first ordering reaches it in 4. Patients with rare conditions or uncommon demographics therefore reach small-cell territory faster than those with common profiles.

To illustrate this disparity hypothetically (not simulated in this study), consider an elderly patient with urosepsis caused by carbapenem-resistant *Klebsiella pneumoniae* being treated with colistin. Each of these attributes (advanced age, urosepsis, a carbapenem-resistant organism, colistin therapy) falls outside the 18 Safe Harbor identifier categories and would survive per-turn de-identification, yet the combination represents a highly distinctive clinical profile; carbapenem-resistant infections account for a small fraction of all *Klebsiella* isolates, and colistin use is restricted to a narrow subset of multidrug-resistant cases. A clinician seeking diagnostic guidance for this patient would disclose a quasi-identifier profile that narrows the anonymity set within a few disclosure steps.

The pattern extends beyond infectious disease. A young adult with BRCA1-associated triple-negative breast cancer on platinum-based adjuvant chemotherapy presents a combination of age decade, germline mutation status, receptor profile, and a non-standard treatment regimen that narrows the anonymity set to single digits within most institutional databases, even though each disclosure individually falls outside the 18 Safe Harbor identifier categories. A pediatric patient with maple syrup urine disease managed on a branched-chain amino acid-restricted formula can reach k=1 at disclosure of the diagnosis alone. Each of these scenarios passes per-turn Safe Harbor review yet cumulatively erodes anonymity within three to four disclosure steps. These trajectories are consistent with our rarity-ordered simulation (median 4 disclosure steps to k<5; Figure 3), where rare clinical attributes drove accelerated decay.

### 4.2 Practical recommendations

#### 4.2.1 On-premise open-source deployment mitigates transmission risk

Local LLM instances (e.g., Llama, Mistral, Qwen via Ollama or vLLM) keep conversation data within institutional infrastructure. However, on-premise hosting does not alter the k-decay inherent in the conversation; if logs are retained, re-identification risk remains for anyone with log access. Institutional controls (access logging, encryption at rest, retention policies) complete the picture.

#### 4.2.2 BAA cloud services reduce risk

Microsoft Azure OpenAI, Google Cloud Healthcare API, and AWS HealthLake provide encryption, access controls, and audit logging. However, BAA coverage addresses contractual obligations; institutions must implement complementary technical controls (network segmentation, encryption of electronic PHI (ePHI) at rest and in transit, and access control and authentication policies) independently (39).

#### 4.2.3 Proposed monitoring solutions require validation

Real-time k-monitoring could alert users approaching small-cell territory, but workflow disruption, alert fatigue, and quality-privacy tradeoffs (limiting disclosure may delay diagnostic support) remain uncharacterized.

#### 4.2.4 Privacy protection does not equal clinical safety

The tension just described assumes the underlying tool is clinically sound, which for general-purpose LLMs is not a given. In 2026, 72% of physicians reported AI use in practice (6), much of it on consumer tools rather than validated medical devices. Under the European Union Artificial Intelligence Act (40), clinical-decision-support AI that qualifies as a medical device under Regulation (EU) 2017/745 (41) is classified as high-risk via Article 6(1) and Annex I; such software must undergo conformity assessment as a medical device. Consumer LLMs such as ChatGPT, Claude, and Gemini are not Conformité Européenne (CE)-marked medical devices, and even LLMs marketed for medical use typically lack the validation required for clinical deployment (10). Parallel expectations exist under U.S. Food and Drug Administration (FDA) guidance on AI-enabled medical devices (42, 43). Within these frameworks, deployment of unvalidated consumer LLMs for clinical decision-making does not satisfy the regulatory expectations described above, regardless of privacy-mitigation choices. On-premise or BAA-covered deployment addresses transmission risk only; responsible governance also requires validated use cases and documented risk-benefit analyses, with regulatory alignment presumed throughout. Independent of regulator action, individual clinicians retain a personal ethical obligation to use validated tools for patient care.

### 4.3 Relation to prior work

Our findings extend prior re-identification research (19, 24) to conversational AI, distinct from training-data extraction (13). Recent work shows LLMs infer personal attributes from public text (44), retain privacy information across sessions (15, 16), and perform autonomous deanonymization at scale (12), while de-identification alone is insufficient for clinical text (14). Our k-anonymity framework quantifies the cumulative disclosure risk.

### 4.4 Limitations

Several limitations warrant consideration. First, we used synthetic data. While this choice reflects an ethical imperative [publishing k-anonymity statistics on real hospital data would itself create attack vectors (31)], Synthea’s conditionally independent attribute generation differs from real EHR correlations. Real patient populations may exhibit different distributions (potentially with stronger comorbidity clustering), yielding different k-decay patterns. Validation studies confirm Synthea produces demographically realistic data (30), but validation on real de-identified datasets (with appropriate Institutional Review Board (IRB) approval and careful consideration of the meta-privacy risks of publishing results) remains an open need. In real clinical populations, correlated comorbidity patterns could affect equivalence class sizes in either direction: common clusters (e.g., diabetes with metformin) may produce larger equivalence classes than independence assumes, while rare comorbidity combinations may produce smaller ones. Several design choices bias toward underestimating k-decay, including age decade binning, single-value disclosure, and omission of laboratory values. However, other factors, including Synthea’s conditional attribute independence and granular ancestry encoding, may have opposing effects. The net direction of these biases is uncertain without validation on real clinical data.

Second, our Progressive Refinement model is grounded in clinical communication conventions but remains theoretical. Real-world prompting may differ: Chief Complaint style prompts often combine multiple attributes in a single turn, and copy-paste workflows would accelerate k-decay beyond our estimates. Because these patterns would likely disclose more information per turn than our model assumes, our progressive refinement model may represent a lower-bound estimate of k-decay rate. Observational characterization of actual clinician-LLM disclosure patterns remains an important direction for future research.

Third, we considered only structured coded data. Natural language processing extraction from free-text clinical notes could identify additional quasi-identifiers, further reducing k. Procedure and allergy data were also simplified to binary indicators (has/has not), which may underestimate k-decay for patients with rare procedures or allergies. Our results may therefore underestimate the extent of privacy erosion in practice. The scope of this limitation extends past our modeling choices. Our Progressive Refinement model reflects physician-style case presentation, whereas a substantial proportion of clinical care is delivered by nursing and allied-health professionals [nurses alone account for roughly 59% of the global health workforce (45)], often using narrative descriptors not fully represented in SNOMED CT. Privacy assessment in these disciplines is also more difficult and more context-dependent than in structured coded data, as narrative descriptors carry contextual information that resists generalization. Our results therefore represent a first step for the structured, physician-facing subset of clinical data and do not generalize directly to unstructured documentation or to the full range of clinical workflows.

Fourth, k values depend on the reference population: a larger reference would yield greater anonymity, but adversaries with auxiliary information or cross-database linkage could still re-identify through methods k-anonymity does not capture.

Fifth, no published cases of patient re-identification from clinical LLM conversations exist; this absence reflects nascent deployment, not absence of risk.

Finally, k-anonymity is necessary but not sufficient; l-diversity and t-closeness address additional vulnerabilities. Specifically, k-anonymity protects against singling-out attacks but is vulnerable to homogeneity attacks, where individuals sharing a quasi-identifier combination share identical sensitive attributes, and to background-knowledge attacks, where auxiliary information enables inference even within an anonymity set (26). L-diversity addresses homogeneity by requiring diverse sensitive-attribute values within each equivalence class; t-closeness further constrains these distributions to approximate the overall population (27). Differential privacy offers a formally quantified complementary framework: calibrated noise injection yields provable bounds (parameterized by ε) on any single individual’s influence (46), with composition theorems that would formally quantify cumulative privacy loss across sequential disclosures (47). In high-dimensional conversational data, homogeneity is common within small equivalence classes; these frameworks therefore complement k-anonymity directly.

Other specific limitations: genomic disclosures may achieve k=1 immediately; we did not analyze disparate impact across subgroups; and multi-session linking was not modeled, though its inclusion would accelerate k-decay.

### 4.5 Future directions

This work motivates research on (1) real-time k-monitoring prototypes, (2) privacy-preserving conversation strategies, (3) automated semantic generalization via clinical ontologies, (4) regulatory guidance on the Minimum Necessary Standard for conversational AI, and (5) observational studies of clinician-LLM disclosure patterns.

### 4.6 Conclusion

These results identify cumulative quasi-identifier exposure as the operational gap in per-prompt de-identification: current frameworks assess each disclosure independently, while consumer AI tools without BAA coverage further expose accumulated information to parties with no contractual obligation to safeguard it. Given physician AI adoption nearly doubling since 2023 (38% to 72%) (6), institutional adoption of on-premise open-source LLMs or BAA cloud services can reduce the adversary pool, while real-time monitoring of cumulative quasi-identifier exposure remains an open need to keep clinical AI aligned with the protections HIPAA was designed to provide.

## Data Availability

The datasets presented in this study can be found in online repositories. The names of the repository/repositories and accession number(s) can be found below: Source dataset: SyntheticMass Version 2 (May 24, 2017) available at https://synthea.mitre.org/downloadsSimulation code and results: GitHub repository at https://github.com/JamesWeatherhead/k-anonymity-decay.
