# Supplementary material for: K-anonymity decay in multi-turn clinical large language model conversations
Source: Front Digit Health. 2026 Jun 25;8:1832168. doi: 10.3389/fdgth.2026.1832168 (PMC13346167; doi:10.3389/fdgth.2026.1832168)
Supplement: Supplementary file 1 [file Datasheet1.pdf]

Supplementary Material

1 SUPPLEMENTARY TABLES

1.1 Table S1: K-Anonymity Decay by Disclosure Step (Progressive Refinement Model)

Table S1. K-anonymity decay by disclosure step (Progressive Refinement model). Based on simulation of 5,000 patients from the 133,262-patient population; percentages calculated among patients reaching each step.

Step	QI(s) Added	Median k	Mean k	% $k < 5$	% $k < 11$	% Unique
1	age decade	13,692	13,852	0.0%	0.0%	0.0%
2	+gender	6,924	6,932	0.0%	0.0%	0.0%
3	+race	4,964	4,224	0.02%	0.02%	0.02%
4	+ethnicity	493	648	0.02%	0.02%	0.02%
5	+marital status	299	444	0.28%	0.80%	0.02%
6	+primary condition	39	80	12.6%	22.7%	4.3%
7	+secondary condition	5	33	49.3%	65.0%	25.4%
8	+primary medication	2	16	70.5%	80.3%	49.0%
9	+has procedure	1	15	72.3%	82.0%	50.9%
10	+has allergy	1	14	75.5%	84.2%	55.7%
11	+first encounter year	1	13	79.9%	86.2%	64.6%

1.2 Table S2: K-Anonymity Decay by Disclosure Step (Rarity-Ordered Model)

Table S2. K-anonymity decay by disclosure step (Rarity-Ordered model, worst-case). QI order varies by patient based on attribute rarity.

Step	Median k	% $k < 5$	% $k < 11$	% Unique
1	1,815	0.06%	0.26%	0.02%
2	152	5.9%	11.9%	1.7%
3	22	25.4%	37.7%	10.6%
4	6	46.5%	59.9%	25.3%
5	2	63.2%	74.6%	40.3%
6	1	72.1%	81.5%	51.6%
7	1	76.7%	84.8%	58.4%
8	1	78.9%	85.9%	62.4%
9	1	79.2%	85.9%	63.3%
10	1	79.4%	86.1%	63.7%
11	1	79.9%	86.2%	64.6%

1.3 Table S3: Complete Quasi-Identifier List

Table S3. Complete quasi-identifiers from SyntheticMass. "Used" indicates inclusion in simulations.

Category	QI Name	Used	Type	Description
Demographics	age decade	✓	String	10-year bins (90+ grouped)
	gender	✓	String	M, F
	race	✓	String	white, black, asian, native, other
	ethnicity	✓	String	Detailed ancestry
	marital status	✓	String	M, S, W, D
Conditions	primary condition	✓	SNOMED CT	Most frequent diagnosis
	secondary condition	✓	SNOMED CT	Second most frequent
	condition codes		Set	All codes (set-valued)
	condition count		Integer	Derivable
	has chronic condition		Boolean	Derivable
Medications	primary medication	✓	RxNorm	Most frequent medication
	medication codes		Set	Set-valued
	medication count		Integer	Derivable
Procedures	procedure codes		Set	Set-valued
	procedure count		Integer	Derivable
	has procedure	✓	Boolean	Procedure indicator
Allergies	allergy codes		Set	Set-valued
	allergy count		Integer	Derivable
	has allergy	✓	Boolean	Allergy indicator
Temporal	first encounter year	✓	Integer	Year of first encounter
	last encounter year		Integer	Less relevant
	encounter count		Integer	Derivable
	care span years		Float	Derivable

1.4 Table S4: Simulation Configuration

Table S4. Simulation parameters for reproducibility.

Parameter	Value
Population size	133,262 patients
Sample size	5,000 patients (3.75%)
Random permutations/patient	30
Total simulation runs	160,000 (Progressive: 5,000; Rarity: 5,000; Random: 150,000)
Thresholds reported	$k < 5$, $k < 11$ (simulation also tracked $k < 20$)
Runtime	3.4 hours
Data source	SyntheticMass v2 (May 2017)

Exact CLI Command for Reproducibility

The analysis was executed using the following command:

```
python main.py \
    --data-dir /path/to/synthea/csv \
    --output-dir results/ \
    --n-permutations 30 \
    --sample-size 5000 \
    --seed 42 \
    --thresholds 5 11
```

Note: The CLI default for `--n-permutations` is 100, but 30 permutations were used for this analysis as specified above. Users wishing to replicate results must explicitly set `--n-permutations 30`.

Data Processing Notes

The SyntheticMass v2 dataset (May 2017) contains malformed rows in certain CSV files. The data loader uses `pandas.read_csv()` with `on_bad_lines='skip'` to exclude rows that cannot be parsed. After removing 197 malformed rows, the final population of 133,262 patients represents the successfully parsed records after loading and merging all relevant tables (patients, conditions, medications, procedures, allergies, encounters).

1.5 Table S5: Verified Exemplar Patient

Table S5. Verified exemplar: Patient 8d112137-37e7-4614-ab38-f233925e0c13

QI	Value	Description
age_decade	60 to 69	Male, 60s
gender	M	Male
race	white	White
ethnicity	french	French ancestry
marital_status	M	Married
primary_condition	15777000	Prediabetes
secondary_condition	40055000	Chronic sinusitis
primary_medication	824184	Augmentin
has_procedure	True	Has procedures
has_allergy	False	No allergies
first_encounter_year	2010	First seen 2010

Table S6. Step-by-step k-collapse for verified exemplar using compound conversational turns. Unlike Tables S1, S2, and S8 which report k after each individual QI disclosure (11 steps), this exemplar groups QIs into compound turns as illustrated in Figure 1.

Compound Turn	Cumulative QIs	k
0	(none)	133,262
1	age decade + gender	8,732
2	+ race + ethnicity	646
3	+ marital status	487
4	+ primary condition	137
5	+ secondary condition	15
6	+ primary medication	2

1.6 Table S7: Distribution of Maximum Disclosure Steps

Table S7. Maximum disclosure steps per patient (n=5,000).

Max Steps	Patients
11	3,631 (72.6%)
10	594 (11.9%)
9	150 (3.0%)
8	625 (12.5%)

1.7 Table S8: K-Anonymity Decay by Disclosure Step (Random Ordering Model)

Table S8. K-anonymity decay by disclosure step (Random Ordering model). Statistics averaged across 30 random permutations per patient (n=5,000 patients; 150,000 permutation runs total). Unlike the Progressive and Rarity-Ordered models which have deterministic QI ordering, the Random model shows the distribution of k-decay under arbitrary disclosure order.

Step	Median k	Mean k	% $k < 5$	% $k < 11$	% Unique
1	29,846	46,746	0.01%	0.03%	0.0%
2	6,714	16,051	0.24%	0.64%	0.06%
3	1,412	5,384	1.7%	3.5%	0.55%
4	359	1,811	5.8%	10.1%	2.1%
5	100	617	13.3%	20.9%	5.6%
6	30	212	24.3%	35.2%	11.8%
7	10	75	37.9%	50.8%	20.5%
8	4	28	52.4%	65.8%	31.7%
9	2	17	65.3%	76.9%	43.9%
10	1	14	74.9%	83.6%	55.6%
11	1	13	79.9%	86.2%	64.6%
